# Epidemiology of adulthood drowning deaths in Bangladesh: Findings from a nationwide health and injury survey

**DOI:** 10.12688/f1000research.10980.1

**Published:** 2017-04-27

**Authors:** Mohammad Jahangir Hossain, Animesh Biswas, Saidur Rahman Mashreky, Fazlur Rahman, Aminur Rahman

**Affiliations:** 1 Centre for Injury Prevention and Research, Bangladesh (CIPRB), Dhaka, Bangladesh; 2 Department of Epidemiology, Bangladesh University of Health Sciences (BUHS), Dhaka, Bangladesh

**Keywords:** fatal, drowning, adult, Bangladesh.

## Abstract

***Background:*** Annual global death due to drowning accounts for 372,000 lives, 90% of which occur in low and middle income countries. Life in Bangladesh exposes adults and children to may water bodies for daily household needs, and as a result drowning is common. In Bangladesh, due to lack of systemic data collection, drowning among adults is unknown; most research is focused on childhood drowning. The aim of the present study was to explore the epidemiology of adulthood drowning deaths in Bangladesh.

***Methodology:*** A nationwide cross-sectional survey was conducted from January to December in 2003 among 171,366 rural and urban households, with a sample of 819,429 individuals to determine the epidemiology of adulthood drowning in Bangladesh.

***Results:***  Annual fatal drowning incidence among adults was 5.85/100,000 individuals. Of these, 71.4% were male and 28.6% were female (RR 2.39). In total, 90% of the fatalities were from rural areas. Rural populations were also found to have a 8.58 times higher risk of drowning than those in urban areas. About 95% of drowning occurred in natural water bodies. About 61.6% of the deaths occurred at the scene followed by 33.5% at the home. Of the drowning fatalities, 67% took place in water bodies within 100 meters of the household. Among the drowning fatalities 78.4% occurred in daylight between 7.00 and 18.00. Over 97% of the victims were from poor socio economic conditions with a monthly income tk. 6,000 ($94) or less. Only 25.5% of incidences were reported to the police station.

***Conclusions:*** Every year a significant number of adults die due to drowning in Bangladesh.  Populations living in rural areas, especially men, were the main victims of drowning. This survey finding might help policy makers and scientists to understand the drowning scenario among adults in Bangladesh.

## Introduction

Drowning is the process of experiencing respiratory impairment from submersion or immersion in liquid, and the outcomes are classiﬁed as death, morbidity and no morbidity ^1^. Drowning is an important but neglected public health issue that affects children and youths in many societies worldwide ^2, 3^. Following road traffic and injury sustained from falls, drowning is the 3 ^rd^ leading cause of injury death in the world, claiming 42 lives every hour and 372,000 lives a year, which is almost two thirds attributed to malnutrition and over half of malaria ^2^. Of all drowning deaths more than 90% occur in low and middle income countries where individuals are exposed to water during daily life ^3– 5^. According to the WHO (2014), drowning contributes to 7% of all injury-related annual deaths worldwide ^6^. South-East Asian countries are considered the most affected region with 2.49 million disability adjusted life years as a result of death and disability from drowning ^7^.

Bangladesh is a low-lying, riverine country located in the subtropical region of South Asia and bordering with the Bay of Bengal. Its tropical monsoon climate is characterized by heavy rainfall and melting snow in the Himalayan territory, leading to large rivers, such as the Ganga, Brahmaputra and Meghna. The country has a landmass of 147,570 square kilometers and is one of the most densely inhabited countries in the world with a population of 160 million. Daily life in Bangladesh exposes people to water bodies, such as ponds, ditches, rivers, canals and the ocean, which are used for daily household needs, including agriculture, fishing and transportation. As a result, drowning effects all ages of the Bangladeshi population.

Most research on drowning conducted in Bangladesh has focused on childhood drowning ^8– 10^. In Bangladesh, there is no established routine mortality registration system ^11^, which, combined with inadequacy of research ^12^, results in unknown drowning deaths among the adult population. To design an appropriate preventive measure for reducing adult drowning, it is important to determine the nationwide burden of drowning. Drowning mostly occurs among the rural populations ^8^, so community-based household survey data is important. The objective of this study was to estimate fatal adult drowning in Bangladesh and its variation by sex, place of residence, and seasonality using a nationally representative survey.

## Methods

Data for this study was extracted from Bangladesh Health and Injury Survey (BHIS), which was conducted during January and December 2003. The following methodology details how the survey data were collected.

### Study design

This was a nationwide community based cross-sectional study.

### Study population

The study population were from 12 randomly selected districts, namely Thakurgaon, Serajgonj, Sherpur, Narsinghdi, Hobigonj, Comilla, Shariatpur, Jessore, Khulna, Pirojpur, Chittagong and Rangamati. The study also covered Dhaka Metropolitan City of Bangladesh. In total, 819,429 individuals were covered in this nationwide study. By using multi-stage cluster sampling technique, a total of 171,366 households were selected; 88,380 form rural areas, 45,183 from district towns and 37,803 from Dhaka Metropolitan city. There are several upazilas (sub districts) in each district. Populations covered in the upazila level was considered as rural population. From each district one upazila was randomly selected. An upazila comprises a number of unions, which is the lowest administrative unit of an upazila, comprising about 20,000 population. From each upazila, two unions were selected randomly and each union was considered as a cluster of this survey. All households in the selected unions were included in the survey. All 12 selected district headquarters and Dhaka Metropolitan City were considered as urban area. In the urban areas, mohalla served as cluster. Mohalla is the lowest part of the city corporation. Each mohalla constituted about 400–500 households. Systematic sampling method was applied to achieve the required number of households.

### Case ascertainment

Individuals 18 years and above who drowned resulting in a fatality were included as a case.

### Data collection and interview

Forty-eight full time data collectors were selected for the data collection and six supervisors were employed for the supervision and monitoring of the data collection process. All data was collected through face-to-face interviews. All selected data collectors and supervisors were trained in collecting data from individuals.

Due to the availability at the household level, mothers were preferred as primary respondent in this survey. However, if the mother was not available, the most knowledgeable members of the household were considered as respondents. Where possible, the head of household and as many members of the household as possible, were present to corroborate or add detail to the respondent’s interview answers. For the identification of any mortality or morbidity cases in the household, screening forms were used. A household member was defined as someone living in the same house, including domestic helpers or long-term guests who shared daily meals and participated in regular activities within the household. For mortality information, respondents were asked about any deaths over the period of last two years, and for morbidity information, respondents were asked about any illness had occurred over the period of last 6 months. If any illnesses/deaths were identified, the interviewer proceeded with further clarification regarding the injuries. Structured questionnaires were used to identify drowning death, and drowning related data was extracted for further analysis. Distance between household of living and drowning site was determined by asking to the respondent, if the site is near to the household then data collector measured it visually. Repeat visits were made to the households where respondents were unavailable during the first visit. In spite of repeated attempts, 2.7% of households could not be interviewed. A total of 166,766 households completed participation in the study.

### Statistical analysis

**Figure 1.**
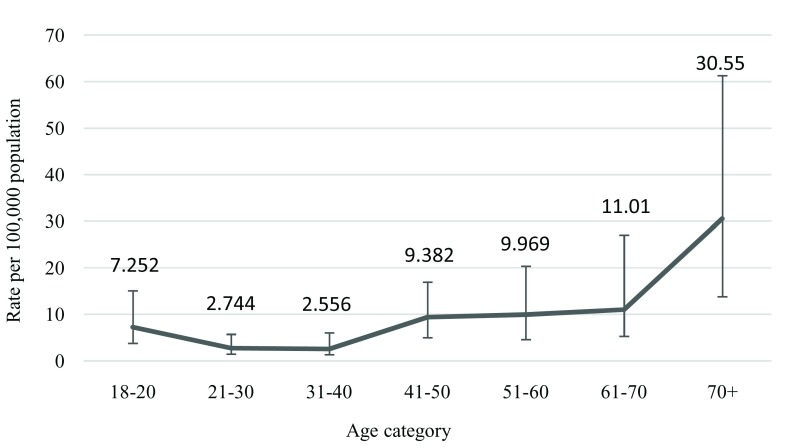
Age wise annual incidence of fatal adult drowning in Bangladesh.

Data related to drowning death were extracted from the main data set. As the recall period was over the last two years, only data from the last year was taken for analysis. Standard descriptive statistics were used to analyze the characteristics of adulthood drowning. Mean, standard deviation (SD), and proportion were used where appropriate. Drowning deaths were presented by gender, age, seasonality and place of residence. Age was categorized into seven groups ( Figure 1). Rates were calculated with 95% confidence intervals (CI). Relative risk (RR) was calculated to compare the drowning risks in different age groups, place of residence, and gender using open EPI-Info software ( http://www.openepi.com/Menu/OE_Menu.htm). The methodology has been described elsewhere ^13– 15^.

## Results

### Incidence of drowning in Bangladesh

In this nationwide cross sectional survey, the annual incidence of drowning fatalities was found to be 5.85/100,000 (95% CI 4.14-8.14) in individuals aged 18 and over. Among the drowning fatalities, 71.40% were male and 28.60% were female. Males were found to be 2.39 times higher at risk than females (RR 2.399; 95% CI 1.04-5.49). Among the victims, 90% were from rural areas and 10% from urban areas. In addition, rural populations were found to have be at an 8.58 times higher risk of drowning than individuals living in the urban areas (RR 8.58; 95% CI 2.47-29.80). The mean age was 46.70 years (SD ± 21.90) ranging from 18 to 95 years. Populations aged over 60 years were found to be 3.60 times higher at risk of drowning compared with the combined populations with ages ranging from 18 to 60 years (RR 3.6; 95% CI 1.14 to 9.15) ( Figure 1 and Table 1).

**Table 1.** Risk factors of fatal drowning among adults in Bangladesh.

Category	Frequency	Rate per 100,000 individuals/year	RR (95% CI)	P value
Sex				
Male	20	8.19	2.39 (1.04-5.49)	0.01
Female	8	3.41	1	
Place				
Rural	25	10.31	8.52 (2.46-29.48)	0.000004
Urban	3	1.2	1	
Age				
60 + years	6	18.12	3.6 (1.14-9.15)	0.005
18–60 years	22	4.94	1	

### Place of drowning

Around 95% of the drowning occurred in natural water bodies, whereas only 5% of fatalities occurred in a place other than a natural water source. About 61.6% of the deaths occurred at the scene followed by 33.5% at the home and 5% in hospital following rescue from water.

### Distance of the drowning site

Of the drowning fatalities, 67% of the incidences took place in water bodies within 100 meters of the household and about 33% of the drowning incidence occurred in water bodies that were over 100 meters of distance from the household.

### Time of drowning

Among the drowning fatalities, 78.4% occurred among in daylight between 07:00 and 18:00, and 21.5% of drowning occurred between 18:00 and 06:00 ( Table 2).

**Table 2.** Details of fatal drowning among adults in Bangladesh (n=28).

Occupation	Frequencies (n)	Percentages (%)
Student	1	2.0
Farmer	5	18.9
Service (working on regular salary)	5	16.5
Daily laborer	5	17.7
Business	1	5.0
Unemployed	9	30.7
House wife	1	4.2
Other	1	5.0
Place of residence		
Urban	3	10.0
Rural	25	90.0
Activities prior drowning		
Bathing	12	44.1
Working (washing clothes)	4	8.9
Travelling (passenger)	2	7.0
Other	4	14.0
Unknown	7	26.0
Time of drowning		
Midnight to 6 am	2	6.2
6 am to 12 noon	11	40.2
Noon to 6 pm	11	38.4
6 pm to midnight	4	15.3
Distance of place of drowning from home		
1 – 5 meters	4	13.9
6 – 10 meters	5	16.5
11 – 20 meters	2	8.9
21 – 50 meters	3	11.2
51 – 100 meters	5	16.5
100+ meters	8	28.4
Unknown	1	4.6
Swimming ability		
Yes	18	62.8
No	7	26.5
Unknown	3	10.7
Place of death		
On spot	17	61.5
Home	10	33.5
Hospital	1	5.0
Reported to the police station		
Yes	7	25.5
No	21	74.5

### Swimming ability

Among the causalities, 62.8% could swim ( Table 2). Swimming ability was defined by reference to ‘‘survival swimming’’ skills (ability to swim 25m) ^16^.

### Seasonality of drowning

**Figure 2.**
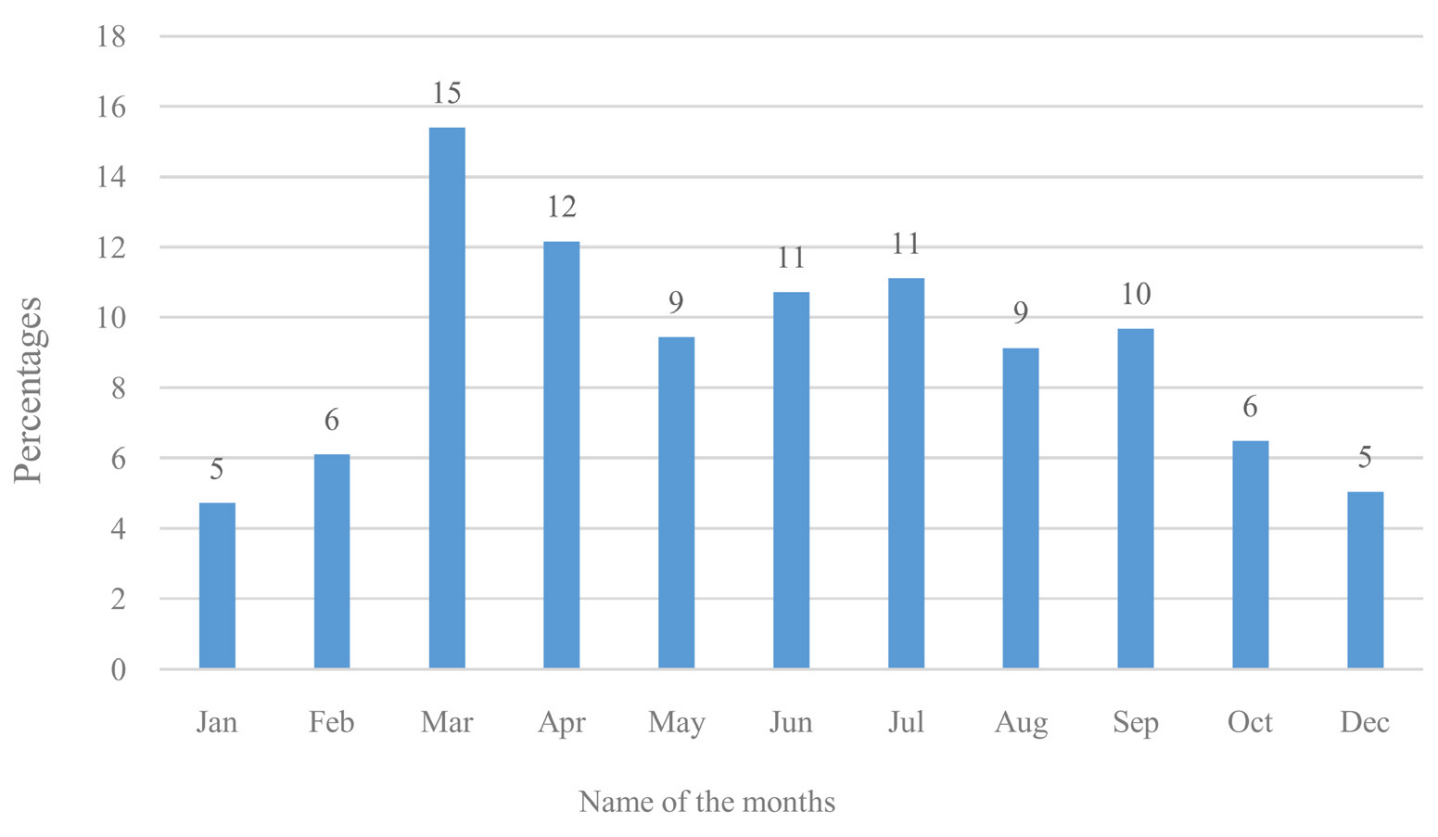
Seasonality of fatal drowning among adults in Bangladesh.

The study findings revealed that drowning incidences were relatively low during the winter season (November to February). The incidence increased during March and September, which are considered as summer and monsoon season. The incidence peaked during March and April ( Figure 2).

### Others factors

Over 97% of the victims were from poor socio economic conditions with a monthly income of tk. 6,000 ($94) or less. Only 25.5% of the incidences were reported to the police station. Among the drowning fatalities, pre-diagnosed individuals with epilepsy and those that were mentally ill totaled 9.6% and 9.9%, respectively.

## Discussion

In Bangladesh, natural and man-made water sources are commonly located in close proximity of households, especially in rural areas. People use these water sources for daily household needs, such as irrigation, fish farming, bathing, swimming, animal feeding and washing clothes. In addition to this, a large number of the population use water transport for regular travelling and goods carrying. As a result, regular exposure to water bodies is very high. Bangladeshi population are experiencing massive destructive natural disasters, such as floods and cyclones, frequently, which often cause a high number of unexpected drowning deaths ( https://en.wikipedia.org/wiki/List_of_Bangladesh_tropical_cyclones). In this study, the main three causes of death due to drowning were bathing, working and travelling.

The survey findings revealed that the annual drowning fatality among adults aged 18 years and above is 5.85/100,000 individuals, which means annually about 8,195 fatal drownings take place among the adult population of Bangladesh. Of these 5,851 are male and 2,344 are female. Adult males were found to be 2.39 times higher at risk of drowning than females in this study. Our findings of higher risk among the male population are similar to other studies on drowning from other countries ^3, 17, 18^.

Individuals aged over 60 years were found to be 3.5 times at a higher risk than those aged between 18 and 60 years. The reasons behind that could be due to lack of a water supply in rural areas; therefore, people use natural water bodies as a source of water for daily regular activities and older populations are not under supervision. Similar findings were also observed in a study conducted among US populations between 1999 and 2010 ^19^.

Drowning is always sudden, unexpected and often fatalities occur at the scene of the water bodies. As a result, drowned individuals need emergency medical support on the site immediately when rescued from the water. Like most developing countries, emergency medical help is absent, particularly in rural areas, of Bangladesh ^20, 21^. In this study, 61.5% of the drowning incidents ended with fatality at the scene of drowning. Findings in Finland suggested that around 24% causalities ended with fatality at the scene ^22^. In addition, of those who were rescued alive (38.5%) from water bodies only 20% sought medical care from the hospital. This suggested that rural populations do not consider receiving medical care following drowning. The study findings show that among the drowning fatalities 56.1% took place in water bodies that were over 20 meters far the household, whereas the same survey finding shows that about 80% of child fatalities due to drowning took place within 20 meters of the household ^23^. In rural Bangladesh, households are located near water bodies so that getting water is easy for daily household needs. As a result exposure to water is very high for both adults and children.

As in most developing countries, injury incidences are poorly reported to the police station by the relatives of the victims ^24^. The survey findings identified that only 25% of cases were reported to the police station following drowning fatalities. Drowning is not a new event concerning injury, like road traffic or machine injury, instead it is an issue that has occurred for thousands of years among populations living near water sources. Rural populations consider drowning as a part of a natural death and pre-decided ‘God’s will ^25^; as a result relatives of the drowning victims start the burial process immediately after fatal drowning occurs. Unless the drowning incident was intentional, relatives of the victim do not report the death to the police station or any other agencies to avoid further investigation about the death.

Many high income countries reduced drowning rates by introducing effective interventions ^1^. This paper describes the epidemiological situation of adulthood drowning in Bangladesh so as to explore people’s perceptions on drowning and to design effective interventions for the adult population further research is needed. In addition, this paper might draw the attention to the policy makers to design possible preventive measures.

## Conclusions

Adult drowning is an important, but neglected, public health issue in Bangladesh, especially in populations living in the rural areas. Every year a significant number of unwanted and preventable adult drowning fatalities occur in Bangladesh. The current survey findings might help policy makers and scientists to understand the epidemiology and the risk factors leading to adult drowning in Bangladesh.

## Data availability

The data referenced by this article are under copyright with the following copyright statement: Copyright: © 2017 Hossain MJ et al.

Data associated with the article are available under the terms of the Creative Commons Zero "No rights reserved" data waiver (CC0 1.0 Public domain dedication). 

BHIS data is stored at the Department of Public Health Science and Injury Prevention of CIPRB. Due to sensitivity of the data (contains identifying information), permission is required from the ethical committee for sharing data with a third party. Data can be requested from the Department of Public Health Science and Injury Prevention of CIPRB, who will contact the ethical review committee to gain approval to share the data. The conditions for gaining data access are a formal request with a clear objective and formal permission from the ethical committee. Please contact Dr Saidur Rahman Mashreky ( mashreky@ciprb.org) in order to request the data.

## Ethics and consent

Ethical approval for the collection of the BHIS data was obtained from the Ethical Committee of the Institute of Child and Mother Health, Dhaka (ref: ICMH/ECR/2002/009). During conduction of the survey all participants were informed about the objectives and benefits of the study. As the sample was over 800,000 individuals, only oral consent was obtained from each of the household head before proceeding the interview.
