# Supplementary material for: Epidemiology of adulthood drowning deaths in Bangladesh: Findings from a nationwide health and injury survey
Source: F1000Res. 2017 Apr 27;6:589. [Version 1] doi: 10.12688/f1000research.10980.1 (PMC5428487; doi:10.12688/f1000research.10980.1)
Supplement: Peer review history

# Peer review: Referee response for version 1

**DOI:** 10.5256/f1000research.11838.r22706

**Published:** 2017-05-15

**Authors:** Puspa Raj Pant

**Affiliations:** 1 Centre for Child and Adolescent Health, University of the West of England, Bristol, UK

Very well composed article, very timely and it must appeal the decision makers and donors. The authors' contribution must be appreciated. As stated by the authors, this paper might draw the attention to the policy makers to design possible preventive measures. Therefore I would like to recommend for its publication. I would like to recommend the authors to look into following minor comments.

1. Introduction, second paragraph: it would be better to add - what proportion of the total area of the country is water i.e. roughly 7% of it is covered with water.
2. Introduction, third paragraph: Would it be possible to highlight the neglected importance of drowning research that the data collected in 2003 that hasn't yet been utilised to uncover the problem of drowning among adult population. Keeping in mind, children related findings were already published with the support of Unicef and other children's agencies.
3. Methods: In the first paragraph, you can also refer previously published Methodological details.
4. Case ascertainment: Please make it clear that the "individuals 18 years and above who drowned resulting in a fatality were included as a case" is for this paper. However, BHIS might have collected much more.
5. Although case identification has been clearly described under "Data collection and interview", the 'mother' is the primary source of information. Therefore there are chances that this study under-reports the adult drowning rates (as compared to GBD estimates are nearly 4 times higher for the year 2003). This can be something that should be considered for adult injuries in future.
6. Discussion: the term 'Regular Travelling' should be replaced by 'Commuting' in the sentence - "a large number of the population use water transport for regular travelling"
7. Discussion: As indicated in #5 above, it is suspected that this study under-reports adult drowning mortality. But the estimates for children are closer to GBD estimates. Is it due to the fact - "In this study, the main three causes of death due to drowning were bathing, working and travelling." or the authors' intention was to say the disaster related drownings for adults were not included.

I have read this submission. I believe that I have an appropriate level of expertise to confirm that it is of an acceptable scientific standard.

# Peer review: Referee response for version 1

**DOI:** 10.5256/f1000research.11838.r22378

**Published:** 2017-05-10

**Authors:** Kazi Selim Anwar

**Affiliations:** 1 Ex: Microbiology Unit, Faculty of Medicine, AIMST (Asian Institute of Medical, Science and Technology) University, Bedong, Malaysia; 2 Institute of Epidemiology, Disease Control and Research (IEDCR), Mohakhali, Dhaka, Bangladesh

**The topic of the article:**

  This is a well written epidemiologically sound and statistically valid original research article on an important topic of public health, titled ‘ Epidemiology of adulthood drowning deaths in Bangladesh: Findings from a nationwide health and injury survey’ highlighting several crucial issues pertaining to drowning epidemiology in the country.

**The quality & merit of this paper:**

 This research paper is based on the analysis of a nationwide cross-sectional survey conducted in Bangladesh in 2003. Data analysis that this study attempted highlights several scientific potentials, public health implications and policy issues. These important findings add values in the current knowledge-base on fatal adult drowning not only in Bangladesh but also in global science - which has the potentiality of being replicated by other researchers/scientists from other countries.

**Overall comment on this manuscript reviewed**

 I recommend this paper for publication in F1000Research but it would definitely carry more value if the authors consider in bringing certain minor changes, as I suggested below:

**The title:**

 It would have sound better as ‘Epidemiology of fatal adult drowning in Bangladesh: Findings from a nationwide health and injury survey’ – it the authors consider only as fine.

**The Methodology part in abstract **(on page 1 of 7):

 Right on the first line of the methodology section, it would have been more rationale to add… ’This updated paper based on a ..’ so as to read the first line of methodology as ‘This updated paper based on a nationwide cross-sectional survey was conducted… again only if the authors consider it as fine **(Thus to make relevant corrections in methodology section on page 3, too)**

**The Introduction: **(on page 3 of 7)

 Even there is no big mistake or major flaw in the introduction section it might have sound more logical & lucid if the authors consider the 2 ^nd^ and 3 ^rd^ paragraph of ‘Introduction’ section to be framed (re-written) as shown below *(first paragraph of introduction looks fine)*:

 Bangladesh is a low-lying, riverine country located in the subtropical region of South Asia and bordering with the Bay of Bengal. The country has a landmass of 147,570 square kilometers being world’s 8 ^th^-most densely populated countries in the world with a population of 160 million people.

 Having a tropical monsoon climate it is characterized by heavy rainfall and melted-snow from the Himalayan territory, leading to three large rivers: the Ganges, the Brahmaputra and the Meghna. Daily life in Bangladesh exposes people to water bodies, such as ponds, ditches, rivers, canals and the ocean- which serve the daily household needs, particularly in rural areas including agriculture, fishing and transportation. In adjunct to country’s geographic & climatic phenomena drowning plausibly effects all ages of the Bangladeshi population compounded by round-the-year prevailing natural disasters like cyclone, flood, hurricane, tidal bore, etc.

 Most of the research conducted in Bangladesh on drowning remains focused on childhood drowning 8–10. In Bangladesh, there is no established routine mortality registration system 11, that also may have contributed in the inadequacy of research 12, resulting in ‘unknown adult drowning deaths’ often among the adult population. To design an appropriate preventive measure in reducing adult drowning, it is imperative to determine the nationwide burden of drowning. Further, drowning mostly occurs among rural populations in Bangladesh 8, so robust data from community-based household survey remains crucial.

 Based on the aforementioned facts & figures, this study was conducted with the objective of estimating gender-specific fatal adult drowning in Bangladesh including seasons and place of residence using the data of a nationally representative survey.

**Data availability: **(on page 6 of 7)

 It is very well referenced, adequately explained and logically utilized (Available data). Only that this section may be taken to methodology section to insert right between 'Statistical analysis' and ‘Results’ sections (on page on page 4 of 7)

**Final comment on the quality of this manuscript reviewed to be published:**

 I strongly recommend that this original article written well scientifically being very sound epidemiologically, pertinent methodologically and valid statistically to be published in F1000Research. This will add values in pertinent topic not only in national (Bangladesh) but also in global data-archiving system towards enhancing the current knowledge base on fatal adult drowning issues.

I have read this submission. I believe that I have an appropriate level of expertise to confirm that it is of an acceptable scientific standard.

# Peer review: Referee response for version 1

**DOI:** 10.5256/f1000research.11838.r22377

**Published:** 2017-05-09

**Authors:** William D. Ramos

**Affiliations:** 1 Department of Recreation, Park, and Tourism Studies, IU School of Public Health-Bloomington, Indiana University Bloomington, Bloomington, IN, USA

The article is timely and relevant. The authors are correct in stating that there is little attention given to adult drowning across the world. This type of epidemiological study is crucial as a first step to developing effective strategies for intervention.

 I would like to see more explanation on how rural versus urban settings were determined as well. In regards to distance of incident to homes, it should be stated that since it was measured visually by data collectors there may be some issues with accuracy.

 More clarification on self-reported swimming ability would also be helpful to better understand the validity of that variable.

 I’m cautious about the use of the Wikipedia source cited in the discussion.

 Overall the article is well developed and methodologically sound. Conclusion drawn appropriately from the data.

I have read this submission. I believe that I have an appropriate level of expertise to confirm that it is of an acceptable scientific standard.
